# Comment on Gentile et al. Superior Survival and Lower Recurrence Outcomes with Breast-Conserving Surgery Compared to Mastectomy Following Neoadjuvant Therapy in 607 Breast Cancer Patients. *Cancers* 2025, *17*, 766

**DOI:** 10.3390/cancers17122008

**Published:** 2025-06-17

**Authors:** Wiebren A. Tjalma

**Affiliations:** 1 Multidisciplinary Breast Clinic and Multidisciplinary Oedema Clinic, Antwerp University Hospital, 2650 Antwerp, Belgium; wiebren.tjalma@uza.be; 2 Faculty of Medicine and Health Sciences, University of Antwerp, MIPRO, 2610 Antwerp, Belgium

The article by Gentile et al. demonstrated that breast-conserving surgery (BCS) in carefully selected patients after neoadjuvant therapy (NAT) is oncologically safe with favorable long-term survival outcomes [1]. The authors emphasize the importance of individualized surgical decision-making to ensure quality of life and safety. While this conclusion is undoubtedly valid, the critical question remains: what criteria should guide patient selection for BCS?

The authors’ table on patient characteristics reveals that 25% of patients did not undergo preoperative mammography, and the percentage who received magnetic resonance imaging (MRI) remains unspecified. According to EUSOMA guidelines, a minimum of 95% (target 98%) of patients with non-metastatic invasive breast cancer should undergo a physical breast examination, mammography, and breast/axillary ultrasound prior to treatment [2]. Furthermore, in cases involving NAT, a minimum of 70% (target 90%) of patients should undergo MRI before and after NAT to properly evaluate the disease extent and pattern for subsequent treatment planning [2].

Ideally, patients should receive comprehensive clinical and imaging reassessment (mammography, ultrasound, and MRI) of both the tumor and axilla following NAT to facilitate individualized surgical decisions. The article by Gentile et al. states that following NAT, all patients underwent either BCS or mastectomy based on tumor response, but the approach to axillary management is notably absent [1]. Current recommendations for post-NAT axillary surgery in patients with cN1 disease converting to ycN0 include targeted axillary dissection or sentinel lymph node biopsy (SLNB) alone [3]. These approaches considerably reduce the morbidity associated with axillary surgery and represent another benefit of NAT.

Several critical elements are missing from the patient characteristics table, including the percentage of patients who underwent mammography, ultrasound, and MRI, as well as the clinical staging (ycTN) after neoadjuvant therapy. The table only presents staging before treatment (cTN) and after surgery (ypTN). An intriguing discrepancy appears in the HER2-positive cohort: 266 patients were HER2-positive, yet 267 patients received trastuzumab, suggesting one HER2-negative patient received anti-HER2 treatment. It would be valuable to examine the distribution of molecular subtypes within the BCS group, as one might expect higher representation of HER2-positive and triple-negative patients, though their higher recurrence rates would likely influence outcomes.

Another methodological issue concerns the use of metallic clips as markers for tumor localization prior to NAT. Why did the authors choose metallic clips rather than hydromarkers? Metallic clips create artifacts on MRI, potentially compromising accurate assessment of tumor response.

For clinical decision-making purposes, it would be informative to know how many patients required conversion from BCS to mastectomy in a second procedure and their corresponding ycTN staging. When counseling patients and their families, understanding the conversion rate is essential for optimal informed consent.

Additional questions arise regarding follow-up protocols: why was MRI not included in surveillance imaging? Also, the inclusion of chest X-rays in follow-up appears to deviate from current guideline recommendations.

The fundamental message from this article confirms that BCS after NAT is feasible and safe, but appropriate patient selection remains crucial. After reading this article, one critical question persist: “How do you select patients?”.
